# *Bacteroides vulgatus* attenuates experimental mice colitis through modulating gut microbiota and immune responses

**DOI:** 10.3389/fimmu.2022.1036196

**Published:** 2022-11-30

**Authors:** Liyun Liu, Mingchao Xu, Ruiting Lan, Dalong Hu, Xianping Li, Lei Qiao, Suping Zhang, Xiaoying Lin, Jing Yang, Zhihong Ren, Jianguo Xu

**Affiliations:** ^1^ State Key Laboratory of Infectious Disease Prevention and Control, National Institute for Communicable Disease Control and Prevention, Chinese Center for Disease Control and Prevention, Beijing, China; ^2^ Research Units of Discovery of Unknown Bacteria and Function, Chinese Academy of Medical Sciences, Beijing, China; ^3^ Department of Epidemiology, Center for Global Health, School of Public Health, Nanjing Medical University, Nanjing, Jiangsu, China; ^4^ School of Biotechnology and Biomolecular Sciences, University of New South Wales, Sydney, NSW, Australia; ^5^ Institute of Public Health, Nankai University, Tianjin, China

**Keywords:** *Bacteroides vulgatus*, colitis, gut microbiota, cytokine, short-chain fatty acids (SCFAs)

## Abstract

**Introduction:**

*Bacteroides vulgatus* is one of the predominant *Bacteroides* species in the human gut and exerts a series of beneficial effects. The aim of this study was to investigate the protective role of *B. vulgatus* Bv46 in a dextran sodium sulfate (DSS) induced colitis mouse model.

**Methods:**

Female C57BL/6J mice were given 3% DSS in drinking water to induce colitis and simultaneously treated with *B. vulgatus* Bv46 by gavage for 7 days. Daily weight and disease activity index (DAI) of mice were recorded, and the colon length and histological changes were evaluated. The effects of *B. vulgatus* Bv46 on gut microbiota composition, fecal short chain fatty acids (SCFAs) concentration, transcriptome of colon, colonic cytokine level and cytokine secretion of RAW 264_·_7 macrophage cell line activated by the lipopolysaccharide (LPS) were assessed.

**Results and Discussion:**

*B. vulgatus* Bv46 significantly attenuated symptoms of DSS-induced colitis in mice, including reduced DAI, prevented colon shortening, and alleviated colon histopathological damage. *B. vulgatus* Bv46 modified the gut microbiota community of colitis mice and observably increased the abundance of *Parabacteroides*, *Bacteroides*, *Anaerotignum* and *Alistipes* at the genus level. In addition, *B. vulgatus* Bv46 treatment decreased the expression of colonic TNF-α, IL-1β and IL-6 in DSS-induced mouse colitis *in vivo*, reduced the secretion of TNF-α, IL-1β and IL-6 in macrophages stimulated by LPS *in vitro*, and downregulated the expression of *Ccl19, Cd19, Cd22, Cd40* and *Cxcr5* genes in mice colon, which mainly participate in the regulation of B cell responses. Furthermore, oral administration of *B. vulgatus* Bv46 notably increased the contents of fecal SCFAs, especially butyric acid and propionic acid, which may contribute to the anti-inflammatory effect of *B. vulgatus* Bv46. Supplementation with *B. vulgatus* Bv46 serves as a promising strategy for the prevention of colitis.

## Introduction

Inflammatory bowel disease (IBD), represented by Crohn disease (CD) and ulcerative colitis (UC), is a disease characterized by chronic gastrointestinal inflammation with relapse remission cycles (1). IBD is a complicated polygenic disorder with multiple etiologies, including genetic susceptibility, environmental factors, impaired gut microbiota (dysbiosis) and mucosal immune response (2). Intestinal flora disorder plays an important role in the pathogenesis of IBD (3). Patients with IBD are associated with a lower microbiota diversity in the gut, in particular, reduced relative abundance of commensal anaerobic bacterial species of the genera *Bacteroides*, *Faecalibacterium*, *Eubacterium* and *Lactobacillus*, are enriched with pathobionts, including *Fusobacterium* and *Escherichia*, compared with healthy controls (4, 5). Hence, regulation of intestinal microbiota is a promising strategy for the treatment of IBD (3).

The Human Microbiome Project established that *Bacteroides* is one of the most dominant genera in the healthy human intestine (6). Patients with IBD, especially in its active phase, tend to have lower levels of *Bacteroides* (7, 8). Thus *Bacteroides* may be marker of gut health, and their loss may be detrimental in the setting of IBD (9). Four *Bacteroides* species, *Bacteroides vulgatus*, *Bacteroides fragilis*, *Bacteroides ovatus* and *Bacteroides thetaiotaomicron*, have been reported to be effective in reducing intestinal inflammatory responses (2, 9–12).

*B. vulgatus* is one of the most prevalent *Bacteroides* species in the human gut microbiota (2, 13, 14). It has been reported that the effects of *B. vulgatus* on IBD were strain specific. Some *B. vulgatus* strains attenuated intestinal inflammatory responses, for example, *B. vulgatus* mpk protected against *Escherichia coli*-induced colitis in interleukin-2-deficient mice by inducing a host anti-inflammatory immune response (11) and *B. vulgatus* 7K1 had protective effect on mouse colitis (12). However, some other *B. vulgatus* strains such as 51K1 and DESEP-B elicited intestinal proinflammatory responses (12, 15). These results suggest that oral administration of different *B. vulgatus* strains have differential effects on intestinal inflammatory responses.

This study investigated the protective effect of *B. vulgatus* Bv46 on dextran sodium sulfate (DSS)-induced mouse colitis model and found that Bv46 could moderate the colitis symptoms, modify intestinal microbiota and alter intestinal immune responses.

## Materials and methods

### Isolation and preparation of *B. vulgatus* Bv46

Freshly emitted fecal samples of healthy individuals were collected as described in our recent study (14). After sample homogenizing and 10-folds serial dilutions, microbial cells in supernatant were plated onto brain heart infusion agar (BHI Agar, BD Difco/BBL, USA) supplemented with 5% defibrinated sheep blood and incubated at 37°C under anaerobic condition. Isolated colonies were subjected to 16S rRNA gene sequencing and the resultant sequences were identified *via* the BLAST program of the EzBioCloud databases. Based on the species demarcation threshold of 98.7% 16S rRNA gene sequence similarity (16), the isolated strain *B. vulgatus* 46 (CGMCC NO.17140) was sub-cultured, harvested and suspended in phosphate buffered solution (PBS) to a concentration of 5×10^9^ colony forming units (CFU)/mL. The *B. vulgatus* Bv46 (0.2 mL/mouse) was administered daily by gavage within 1 h after harvest. OD_600_ measurements and viable counts were used to confirm the concentration of bacteria.

### Genome sequencing and comparison

The genomic DNA of *B. vulgatus* Bv46 extracted using the Wizard Genomic DNA Purification kit (Promega) was sequenced on the Illumina HiSeq TM2000 platform, and the draft genome was assembled as described previously (17). Genome sequence of fourteen other *B. vulgatus* strains (12) were retrieved from NCBI. All the genomes were annotated using Prokka v1.14.6 (18).

To investigate the phylogenetic relationships and evaluate the genetic distance among *B.vulgatus* strains, orthoFinder v2.5.4 was used to cluster orthologous genes (19), and MAFFT v7.453 was used for constructing multi-genome alignments (20), a neighbor-joining tree was then constructed with the maximum composite likelihood method with GAMMA distributed correction on rates among sites using MEGA X (21). The alignment gap or missing data were treated as pairwise deletion. The branches were evaluated using 1,000 times bootstrap resampling. Pan-genome computation of *B.vulgatus* strains Bv46, ATCC 8482, mpk and FTJS7K1 was performed using Roary (22), unique genes of *B.vulgatus* Bv46 were assigned a functional Clusters of Orthologous Group (COG). To predict the carbohydrate unitization, carbohydrate-active enzymes (CAZymes) were identified by searching for matches in the CAZy database using the standalone tool dbCAN v3.0.7 (23).

### Colitis model

Specific-pathogen free (SPF) female C57BL/6J mice (6 weeks old) weighting 16-18 g were obtained from Beijing Vital River Laboratory Animal Technology. After a one-week acclimation, the mice were randomly divided into three groups (n = 8/group): control, PBS-treated colitis (DSS group) and *B. vulgatus* Bv46*-*treated colitis group (BV group). Both the DSS and BV groups were fed 3% DSS (36-50kDa, S0798, MP Biomedicals Canada) for 7 consecutive days to induce colitis. Meanwhile, *B. vulgatus* Bv46 (1×10^9^ CFU/mouse) and PBS were given orally in the BV and DSS groups, respectively. Control group was given plain water. The experiment mice were monitored daily for body weight, stool consistency and rectal bleeding, and the disease activity index (DAI) was calculated as previously described (24). At the end of the experiment, fresh feces were collected, frozen in liquid nitrogen, and then stored at -80 °C refrigerator. After the mice were euthanized, the colons were collected for histopathological observation, and the cecal contents were collected for microbiota analyses. The colon lengths from the ileocecal junction to the anus were measured (25).

### Histopathology analysis of the colon

The distal colon segment of each mouse was fixed in 4% paraformaldehyde (PFA) (BL539A, biosharp, China) for histological analysis. Cross sections were stained with hematoxylin-eosin (H&E) and the pathological features were analyzed by two qualified histopathologists without knowledge of classification. Histopathological analysis was performed according to the colitis scoring system (2, 9) with scores ranging from 0 (no pathology) to 4 (highest pathology). The average percentage of field was calculated at a given score and compared between treatment groups.

### 16S rRNA gene sequencing and compositional analysis

Bacterial genomic DNA was extracted from mouse cecal contents using MagPure Stool DNA KF kit (Magen, China), and the V3-V4 hypervariable regions of prokaryotic 16S rDNA were magnified with universal primers 341F (5’ -ACTCCTACGGGAGGCAGCAG-3’) and 806R (5’-GGACTACHVGGGTWTCTAAT-3’). After quality assurance, equimolar amounts of validated amplicon libraries were sequenced on Illumina HiSeq 2500 platform by BGI Tech Solutions Co., Ltd (Beijing, China) following the manufacturer’s instructions. The resulting paired-end reads were analyzed using scripts of EasyAmplicon v1.14 (26). For β-diversity analysis, Bray-Curtis dissimilarity matrice was measured and used in a non-metric multidimensional scaling (NMDS) analysis.

To determine potential microbial biomarkers in three groups, Linear Discriminant Analysis Effect Size (LEfSe) was calculated using the online tool (27). For the quantification of *B. vulgatus* in feces, species specific real-time PCR was performed as previously described (28, 29). Microbiota sequencing data used in this study are available at https://www.ncbi.nlm.nih.gov/bioproject/PRJNA872285.

### Colons transcriptome analysis

Total RNA was extracted using TRIzol reagent (Invitrogen, Carlsbad, CA) according to the manufacturer’s instructions and genomic DNA was removed using DNase I (Qiagen, Valencia, CA). The RNA size, integrity, and total amount were measured using a Bioanalyzer 2100 (Agilent Technologies, Santa Clara, CA).

As described previously (30–33), RNA sequencing (RNA-seq) transcriptome libraries were prepared using TruSeqTM RNA sample preparation Kit (Illumina, San Diego, CA) according to the manufacturer’s protocol and sequenced on the Illumina sequencing platform (HiSeq 4000). RNA-Seq was performed by BGI Genomics Co., Ltd.

The raw paired end reads were trimmed and quality controlled by Trimmomatic with default parameters (34). Clean reads were then subjected to Salmon (35) in mapping-based mode for transcript quantification, and the current version of Mus_musculus (http://ftp.ensembl.org/pub/release-106/) was served as a reference. Differentially expressed genes (DEGs) between the BV and DSS groups were subsequently identified using the DESeq2 package (36), the thresholds were set as: adjusted *P* value < 0.05, log2(fold change) > 1.0 or log2(fold change) < -1.0. To further understand the biological roles of colonic DEGs, Gene Ontology (GO) enrichment analysis was conducted using clusterProfiler package (37), the enriched maps of GO biological processes were visualized using Cytoscape v3.7.2. For Kyoto Encyclopedia of Genes and Genomes (KEGG) pathway analysis, the gene set enrichment analysis (GSEA) (38) was applied to pre-ranked colon genes based on the log2 transformed fold change in expression, gene sets with the false discovery rate (FDR) < 0.01 and the absolute value of normalized enrichment score (|NES|) > 1.0 were defined as statistically significant. RNA-seq data are publicly available at NCBI under accession number PRJNA872866.

### Reverse transcription quantitative real-time PCR

Reverse transcription quantitative real-time PCR (qRT-PCR) was performed to validate five selected DEGs (*Cd40*, *Cd19*, *Cd22, Ccl19* and *Cxcr5*) identified from transcriptome sequencing. qRT-PCR primers were listed in **Table S1** in the **Supplemental Material**.

Total RNA extraction was performed using TRIzol reagent (Invitrogen, Carlsbad, CA) following the manufacturer’s instructions, and cDNA synthesis was conducted using the PrimeScript™ RT Reagent Kit (Perfect Real Time, TaKaRa). qRT-PCR was carried out using SYBR Premix Ex Taq II (Perfect Real Time; TaKaRa) in a Rotor-Gene Q thermal cycler (Qiagen, Valencia, CA). Data were analyzed with Rotor-Gene Q series software version 1.7 (Qiagen, Valencia, CA). The data were normalized to the endogenous reference gene *β-actin* and analyzed by the cycle threshold method (2^-ΔΔCt^) (39). Three independent replicates were carried out for each target.

### Quantification of colonic inflammatory cytokines in the DSS and BV groups

Colon samples weighing 0.1g were homogenized in PBS and then centrifuged at 12000 g (10 min at 4°C). The amount of tumor necrosis factor (TNF)-α, IL-6 and IL-1β in the supernatant of the colon homogenates were determined by enzyme-linked immunosorbent assay (ELISA).

### Evaluation of anti-inflammatory effects of *B. vulgatus* B46 on macrophages *in vitro*

The anti-inflammatory effect of *B. vulgatus* Bv46 on the lipopolysaccharide (LPS, L2880, Sigma-Aldrich) activated RAW 264_·_7 macrophage cell line was tested as described previously (40). After 48 h of anaerobic culture in BHI broth at 37°C, *B. vulgatus* Bv46 was harvested and suspended in PBS at a concentration of 1×10^8^ CFU/mL. Then the *B. vulgatus* Bv46 live bacteria were added to RAW 264_·_7 cells (multiplicity of infection, MOI = 20) for 6 or 20 h in the presence of 1 μg/mL LPS. At the same time, cells treated with 1 μg/mL LPS were used as positive control, and the non-treated cells as negative control. TNF-α, IL-1β and IL-6 levels of the cell supernatants were determined by ELISA.

### Quantification of short chain fatty acids

After 48 h anaerobic incubation in BHI broth at 37°C, 0.1 mL supernatant of pure culture *B. vulgatus* Bv46 was collected, and then added to a mixture containing 0.05 mL of 50% H_2_SO_4_ and 0.2 mL of methylvaleric acid. The solution was swirled for 1 min and ultrasonically treated for 10 min (incubated in ice water). After centrifugation for 15 min at 9600 g, 4°C, the supernatant was transferred into a fresh vial for gas-chromatography tandem mass spectrometry (GC-MS) analysis.

For fecal samples, 150 mg feces was added to 1 mL H_2_O, homogenized in a ball mill for 4 min at 45 Hz, and then ultrasonically treated for 5 mins (incubated in ice water). After centrifugation for 20 min at 5000 rpm, 4°C, 0.8 mL of the supernatant was collected and transfered to the mixture containing 0.1 mL of 50% H_2_SO_4_ and 0.8 mL of methylvaleric acid. Subsequently, the solution was vortexed (10 s), sonicated (10 min, ice water bath), and centrifuged (12,000 g, 15 min, 4°C), then the obtained supernatant was transferred to a fresh glass vial for GC-MS analysis. A Shimadzu GC2030-QP2020 NX gas chromatography-mass spectrometer (Kyoto, Japan) fitted with a HP-FFAP capillary column (30m × 250 μm × 0.25 μm, J&W Scientific, Folsom, CA, USA) was used for short chain fatty acids (SCFAs) quantification as described previously (41).

### Statistical analysis

Statistical analysis was conducted using GraphPad Prism 9.0. Data presented as mean ± standard deviation (SD). The unpaired Student’s t-test was employed to compare two groups. One-way analysis of variance (ANOVA) followed by the Tukey multiple comparison test was applied to datasets comparing more than two groups. *P* < 0.05 was considered statistically significant.

## Results

### *B. vulgatus* Bv46 exhibits strain-specific characteristics

To investigate the genomic differences between *B. vulgatus* Bv46 and other *B. vulgatus* strains, we conducted a comparative genome analysis. A neighbor-joining phylogenetic tree based on 1796 core genes was constructed, which showed that *B. vulgatus* Bv46 was placed in its own lineage that was sister to strain FJSWX62K35, and separated from *B. vulgatus* strains ATCC 8482, mpk and FTJS7K1, all of which have been reported to attenuate colitis (**Figure S1A**). Comparison of *B. vulgatus* strain Bv46 with ATCC 8482, mpk and FTJS7K1 showed that *B. vulgatus* Bv46 had 872 unique genes. Functional annotation assigned the *B. vulgatus* Bv46 strain-specific genes to 21 COG classes, with the majority in replication, recombination and repair (L, 44 genes), cell wall/membrane/envelope biogenesis (M, 40 genes), transcription (K, 23 genes), and carbohydrate transport and metabolism (G, 19 genes) apart from genes with unknown function (**Figures S1B, Table S2**). Further carbohydrate-active enzymes analysis predicted that the unique genes of *B.vulgatus* Bv46 contained 25 glycosyltransferases (GTs) genes, 12 glycoside hydrolases (GHs) genes, 1 carbohydrate-binding module gene and 1 polysaccharide lyase gene (**Table S3**).

### *B. vulgatus* Bv46 ameliorated DSS-induced colitis in mice

**Figure 1:**
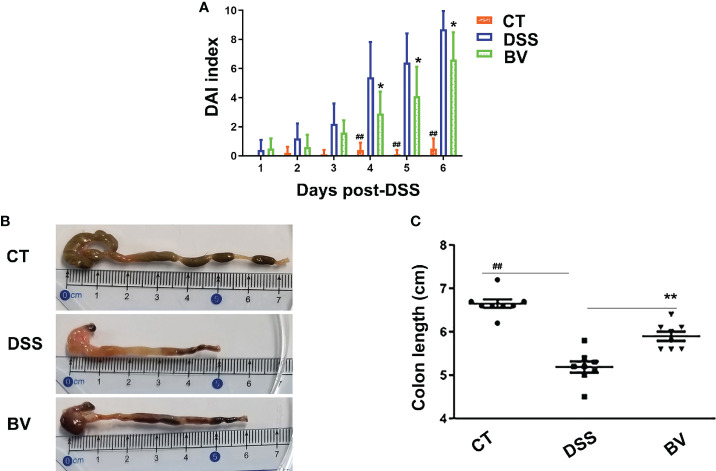
Effect of *B. vulgatus* Bv46 on the DSS-induced colitis in C57BL/6J mice. Disease activity index (DAI) scores **(A)** and colon length **(B, C)** of the DSS-induced mice treated with *B. vulgatus* Bv46 (in the BV group), or phosphate buffer saline (in the DSS group), and control (CT, no DSS induction) for 7 days. Values represent the mean ± standard deviation. *, *P* < 0.05 and **, *P* < 0.01 indicated the significant differences of the DSS versus BV group; ^##^, *P* < 0.01 indicated the significant differences of the DSS versus control group.

To test whether *B. vulgatus* Bv46 is protective against DSS-induced colitis in mice, we determined the severity of colitis by monitoring the DAI index during the 7-day induction period and the colonic length at the end of experiment. As shown in **Figures 1** and **S2**, the DSS group displayed notable weight loss (days 4-7 of DSS exposure; *P* < 0.01 versus control group), high DAI score (days 4-6 of DSS exposure; *P* < 0.01 versus control group) and shortened colon length (at day 7 of DSS exposure; *P* < 0.01 versus control group). Comparing with the DSS group, the DAI score was significantly reduced in the BV group (*P* < 0.05 at days 4-6; **Figure 1A**), and the colon length was significantly longer in the BV group at day 7 of DSS exposure (*P* < 0.01; **Figures 1B, C**). However, the weight losses were comparable between the DSS and BV groups (*P* > 0.05; **Figure S2**).

**Figure 2:**
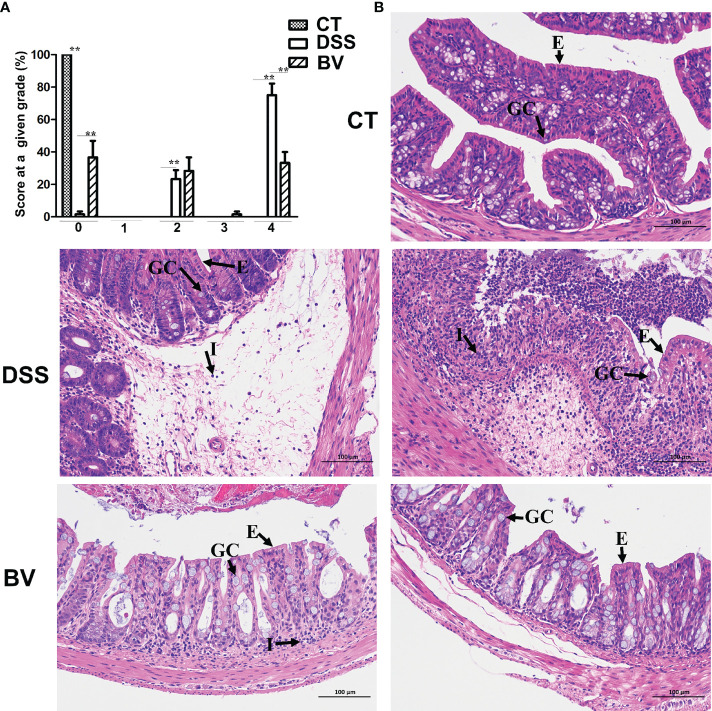
Histopathology of *B. vulgatus* Bv46 effect on acute DSS-induced colitis in C57BL/6J mice. **(A)** Histopathological scores. **(B)** Representative images of H&E stained sections of ascending colons from indicated groups; GC: goblet cell; E: epithelium; I: cellular infiltrate. CT, DSS, and BV denote control (no DSS induction), DSS induction and treatment with phosphate buffer saline only, and DSS induction and treatment with *B. vulgatus* Bv46, respectively. **, P < 0.01.

Microscopic examination of colon histopathological sections showed that DSS-induced histopathological damage was generally moderate to severe, and mucosal lesions and submucosal inflammatory cell infiltration were observed in some visual fields. In the DSS group, 23.3% visual fields showed moderate scores (grade 2), 75% visual fields showed high scores (grade 4) and only 1.7% visual fields with no pathology (grade 0) (**Figure 2A**). In the control group, 100% visual fields had no pathology (grade 0). There were significant differences in pathological grades 0, 2 and 4 between the control and DSS group (*P* < 0.01). In the BV group, 36.7% visual fields had no pathology (grade 0), 28.3% visual fields had moderate pathology (grade 2) and 33.3% visual fields had high pathology (grade 4). Compared with the DSS group, histopathological scores in grades 0 and 4 were significantly reduced (*P* < 0.01) in the BV group (**Figure 2A**). Inflammatory cell infiltration, mucin depletion and epithelial erosion were all less evident in the BV group (**Figure 2B**).

### *B. vulgatus* Bv46 modified the mouse gut microbiota community

**Figure 3:**
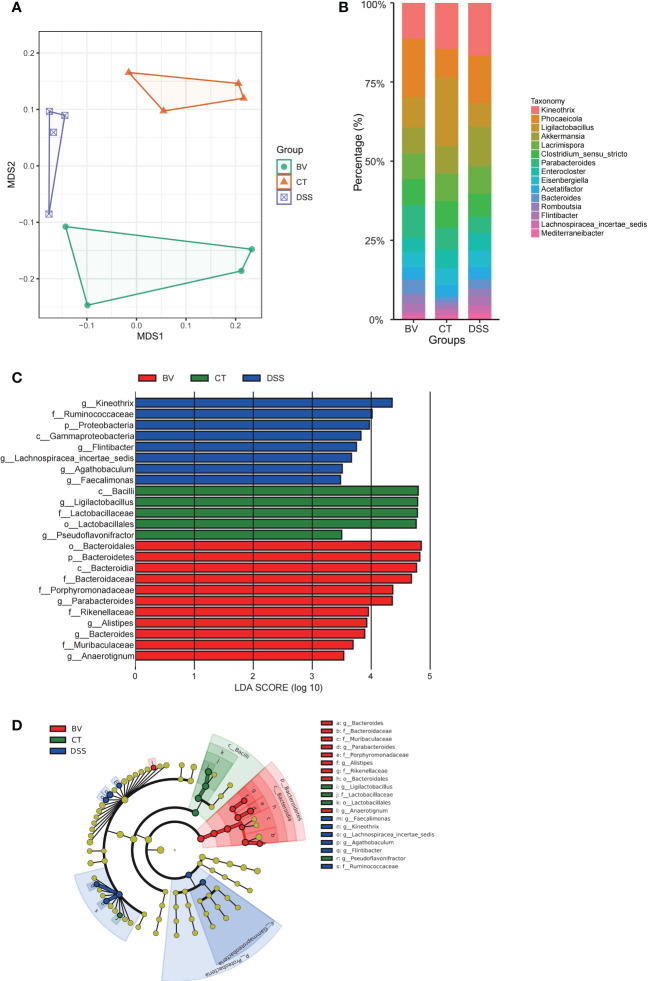
Effect of *B. vulgatus* Bv46 on intestinal microbiota composition in DSS-induced colitis mice. CT, DSS, and BV denote control (no DSS induction), DSS induction and treatment with phosphate buffer saline only, and DSS induction and treatment with *B. vulgatus* Bv46, respectively. **(A)** Ordination of microbial profiles using non-metric multidimensional scaling (NMDS) plotting. **(B)** Barplot analysis of the microbial compositional profiling at the genus level (top 15). **(C)** Overrepresented bacterial taxa among groups determined by Linear discriminant analysis (LDA). **(D)** A cladogram of taxa shown in **(C)** (from phylum to genus).

The V3-V4 region of the 16S rDNA was sequenced to assess the modulatory effects of *B. vulgatus* Bv46 on gut microbiota of the DSS-induced mice. The rarefaction curves of individual groups indicated that the sequencing depth was adequate to reliably describe the bacterial microbiome associated with these three groups (**Figure S3A**). NMDS analysis showed that the gut microbiota profiles (β diversity) were grouped according to diet (**Figure 3A**). The top abundant taxa at the phylum and genus levels were shown in **Figures 3B** and **S3B**. Similar to previous studies (42), at the phylum level, Firmicutes and Bacteroidetes were the predominant constituents in mouse gut microbiota (**Figure S3B**). **Figure 3B** showed the top 15 abundant genera among the control, DSS and BV groups. In the control group, *Ligilactobacillus* had the highest relative abundance. However, in the DSS group, the relative abundance of *Ligilactobacillus* was obviously lower, and *Kineothrix* was the dominant genus. In the BV group, *Phocaeicola* emerged as a dominant genus.

To identify bacterial taxa that have changed significantly among the control, DSS and BV groups, colonic microbial composition was analyzed the LEfSe based on nonparametric factorial Kruskal-Wallis and Wilcoxon tests. **Figures 3C, D** showed the taxa with significant differences, indicated by an LDA score greater than 3.0, which reflected the degree of influence of a taxa with a significant difference among the three groups. At the phylum and class levels, Bacteroidetes and *Bacteroidia* were enriched in the BV group. Moreover, *B. vulgatus* Bv46 treatment significantly increased the abundance of *Parabacteroides*, *Bacteroides*, *Anaerotignum* and *Alistipes* at the genus level. For the DSS group, genera *Kineothrix*, *Agathobaculum*, *Faecalimonas* and *Flintibacter* were enriched. However, genus *Ligilactobacillus* was enriched in the control group. With respect to *B. vulgatus*, a higher biomass was observed in the feces of mice fed with *B. vulgatus* Bv46, compared with that of the DSS group (**Figure S3C**).

### *B. vulgatus* Bv46 regulated the immune signaling pathways

**Figure 4:**
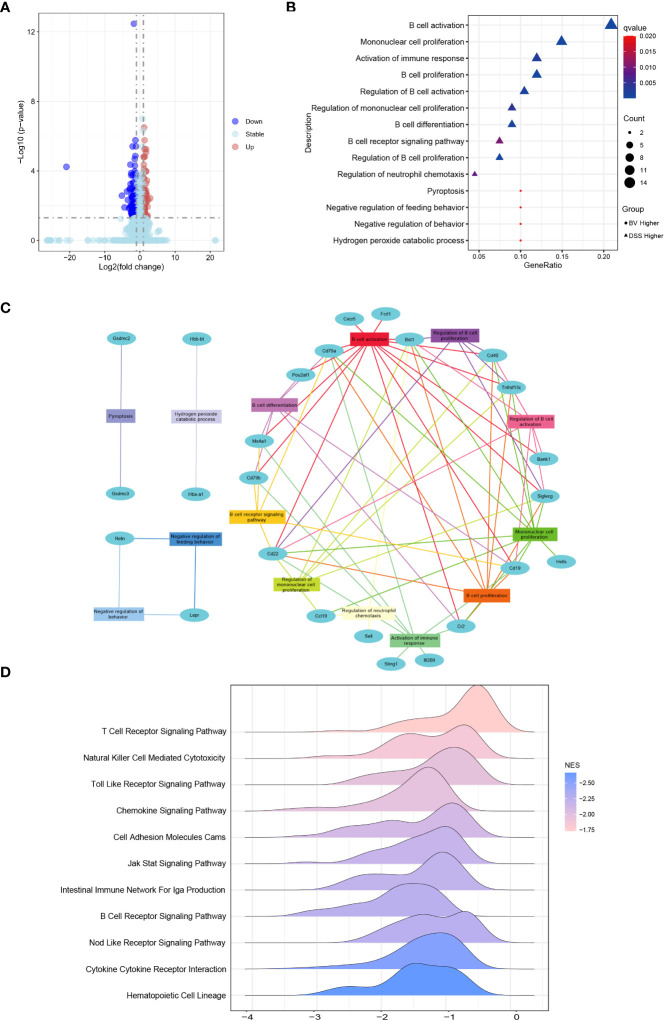
Effects of *B. vulgatus* Bv46 on transcriptome profiling and functional analysis under the IBD setting. CT, DSS, and BV denote control (no DSS induction), DSS induction and treatment with phosphate buffer saline only, and DSS induction and treatment with *B. vulgatus* Bv46 respectively. **(A)** Volcano plots analysis for the distribution of colonic differentially expressed genes (DEGs) between the BV and DSS groups. **(B)** Functional enrichment analysis of colonic DEGs in the BV vs DSS groups. GO biological processes enriched in the up-regulated and down-regulated DEGs are plotted in triangle and circle, respectively. **(C)** Network analysis of colonic DEGs and the enriched GO biological processes. **(D)** *B.vulgatus* Bv46 induced changes of immune related KEGG pathways identified by the gene set enrichment analysis (GSEA) in colons. The distribution of core enriched genes was plotted based on fold changes on the horizontal axis.

Colonic RNA-seq was performed to analyze the differential expression of colonic genes between the DSS and BV groups. There were 25 upregulated and 83 downregulated genes in the BV group compared to those in the DSS group (**Figure 4A**). Through GO-BP (Biological Process) enrichment analysis, 19 downregulated DEGs in the BV group were significantly enriched in the regulatory B cell response, such as B cell activation, proliferation and differentiation pathway, B cell receptor signaling pathway and regulation of neutrophil chemotaxis pathway; two upregulated DEGs (*Hba-a1* and *Hbb-bt*) in the BV group were significantly enriched in hydrogen peroxide catabolic process (**Figure 4B**, **Table S4**). According to the BP pathway analysis, the downregulated genes, *Cd40*, *Cd79a*, *Cd19*, *Cd22*, *Siglecg*, *Bst1* and *Tnfrsf13c* participated in over six pathways (**Figure 4C**, **Table S5**).

**Figure 5:**
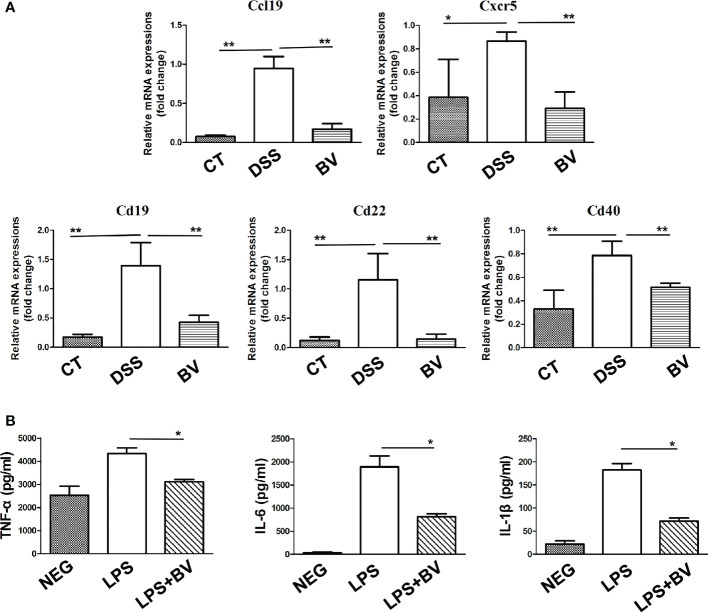
Effect of *B. vulgatus* Bv46 on the expression of *Ccl19, Cd19, Cd22, Cd40* and *Cxcr5* genes in mice colon and the secretion of TNF-α, IL-1β and IL-6 in macrophages stimulated by LPS *in vitro.* CT, DSS, and BV denote control (no DSS induction), DSS induction and treatment with phosphate buffer saline only, and DSS induction and treatment with *B. vulgatus* Bv46 respectively. **(A)** Effects of *B. vulgatus* Bv46 administration on the expression of inflammatory genes in DSS-induced colitis mice. Mouse colons were collected and the mRNA expression of *Ccl19*, *Cxcr5*, *Cd19, Cd22*, *Cd40* was quantified using real-time PCR. Fold changes are expressed as mean ± SD (*n* = 3 each group). *P < 0.05, **P < 0.01. **(B)** The cytokine profiles (concentrations of TNF-α, IL-1β and IL-6) of the supernatants after the cultivation of untreated RAW264.7 cells or cells after treatment with LPS or LPS+BV. Error bars are SD. *P < 0.05 for LPS+BV versus LPS. NEG, negative control.

Further GSEA analysis of mice colon transcripts found that 12 upregulated and 34 downregulated KEGG pathway terms were enriched in the BV group (**Table S6**). Among 34 downregulated KEGG pathway terms, 11 were immune-related signaling pathways, including T cell receptor signaling pathway, Toll like receptor signaling pathway, cell adhesion molecules, B cell receptor signaling pathway and cytokine-cytokine receptor interaction (**Figure 4D**). Five of 19 downregulated DEGs in the BV group, including *Cd40*, *Cd19*, *Cd22, Ccl19* and *Cxcr5* also partake in two or more immune-related GSEA KEGG signaling pathways (**Table S5**). To further validate the RNA-Seq data, these five genes were confirmed by qRT-PCR, which exhibited a concordant direction in both RNA-Seq and qRT-PCR (**Figure 5A**).

### *B. vulgatus* Bv46 affected the expression of colonic inflammatory cytokines

The expression of colonic inflammatory cytokines TNF-α, IL-6 and IL-1β in the DSS and BV groups were determined. The expression of these three colonic proinflammatory cytokines in the BV group were lower than those in the DSS group (*P* < 0.05; **Figure S4**).

### *B. vulgatus* Bv46 modified cytokine secretion *in vitro*

The macrophage RAW 264.7 was used to evaluate the potential anti-inflammatory effect of *B. vulgatus* Bv46 (40, 43, 44). As showed in **Figure 5B**, *B. vulgatus* Bv46 markedly reduced the secretion of TNF-α, IL-1β and IL-6 in LPS-stimulated RAW 264.7 cells (*P* < 0.05).

### Production of SCFAs by *B. vulgatus* Bv46 and fecal SCFAs content

Five SCFAs, including acetic acid, propionic acid, butyric acid, isobutyric acid and isovaleric acid were detected in the culture supernatant of *B. vulgatus* Bv46 with concentrations of 1486.21, 173.75, 0.95, 6.07 and 8.15 μg/ml, respectively.

**Figure 6:**
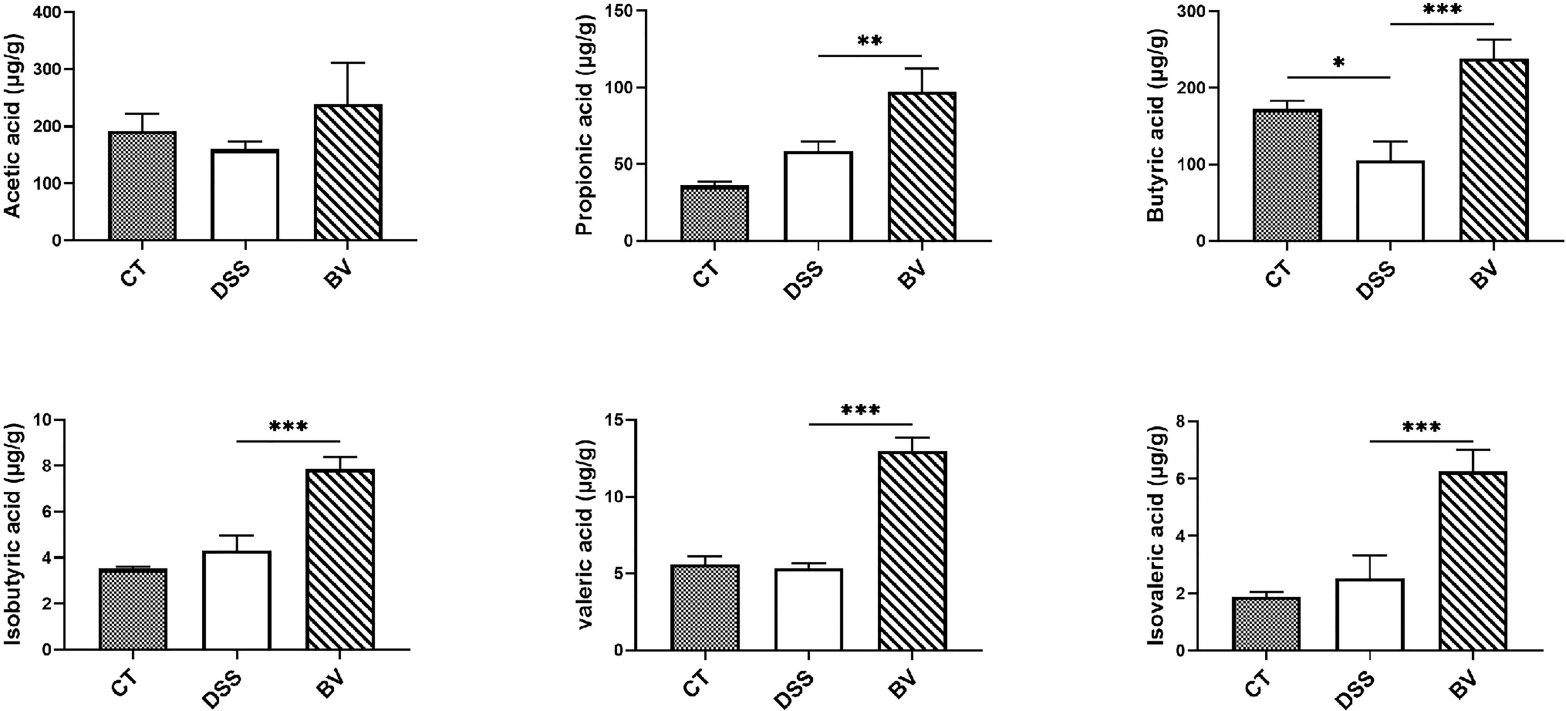
Comparison of fecal short chain fatty acids (SCFAs) profiles. Statistical comparison was implemented by a one-way ANOVA followed by Tukey’s multiple-comparison test. Values represent the mean ± standard deviation. Significance levels are shown as *P < 0.05, **P < 0.01, ***P <0.001.

As shown in **Figure 6**, the fecal level of acetic acid, butyric acid, propionic acid, isobutyric acid, valeric acid and isovaleric acid were determined for all groups. Excepting for butyric acid, there was no significant difference in fecal SCFAs between the CT and DSS groups. The BV group showed significantly higher concentration of five of the six fecal SCFAs (except for acetic acid) compared with the DSS group (*P* < 0.05), indicating that *B. vulgatus* Bv46 supplementation contributed to the increase of these SCFAs.

## Discussion

*B. vulgatus* is one of the predominant *Bacteroides* species in the human gut, and it has been reported that *B. vulgatus* has beneficial effects on the host (2, 13, 14). Notably, *B. vulgatus* improved intestinal inflammatory responses in mouse colitis depending on the *B. vulgatus* strain used (11, 12, 45). In this study, we demonstrated that *B. vulgatus* Bv46 isolated from a fecal sample of a healthy volunteer has protective effects on the primary efficacy readouts of colitis.

In previous studies, it has been demonstrated that some *Bacteroides* species have protective effects on animal models of colitis (2, 9, 11, 12, 45). For example, *B. thetaiotaomicron* DSM 2079 displayed strong protective effects on DSS-induced mice and rat colitis (2). *Bacteroides ovatus* ATCC 8483 monotherapy and triple-*Bacteroides* combination (*B. ovatus*, ATCC 8483B. *vulgatus* ATCC 8482 and *B. thetaiotaomicron* ATCC29148) had protective effects on a mouse colitis model, and *B. ovatus* was superior to multi-strain bacterial therapy (9). *B. vulgatus* 7K1 was shown to be potentially efficacious for alleviating DSS-induced colitis and lipopolysaccharide-induced acute intestinal injury in mice (12, 45). The phylogenetic analysis of 15 *B. vulgatus* strains showed that *B. vulgatus* Bv46 was significantly different from the other three strains, ATCC 8482, mpk and FTJS7K1, that showed probiotic potential. Based on comparative genome analysis, strains-specific genes of *B. vulgatus* Bv46 were found to mainly encode specific proteins related to glycosyl transferases (GTs) and glycoside hydrolases (GHs), followed by carbohydrate-binding modules (CBMs) and polysaccharide lyases (PLs). GHs are involved in catalyzing the breakdown of plant-derived oligo- and poly-saccharides to fermentable monosaccharides, and the major products of fermentation are SCFAs (46), which provide nutrients to the colonic epithelium and exert anti-​inflammatory effects (47). In *B. vulgatus* Bv46, the strain-specific genes encoding GT2 and GT4 were found to be abundant, these gene families typically play a role in lipo- and/or exopolysaccharide biosynthesis essential for signaling process or cell wall polysaccharide synthesis (48, 49). Our results support that the probiotic effect of *B. vulgatus* is strain specific as our strain is well separated phylogenetically from the other strains that show similar protibiotic effect. They may share similar mechanisms of protection with functionally equivalent genes. Further functional studies will be required to elucidate the genes or pathways involved.

To maintain intestinal health, it is necessary to promote the growth of beneficial bacteria and minimize the pathogenic bacteria in the intestinal flora (50). Our results showed that *B. vulgatus* Bv46 treatment significantly increased the abundance of *Parabacteroides*, *Bacteroides*, *Anaerotignum* and *Alistipes*. *Parabacteroides*, *Bacteroides* and *Alistipes* are considered the main candidates for next-generation probiotics, which have attracted considerable attention due to their anti-inflammatory properties (9, 40, 51). *Parabacteroides* has protective effects against colitis, multiple sclerosis, epilepsy, obesity, metabolic dysfunctions, and tumors (40, 52–55). *Parabacteroides distasonis* attenuated IL-8 release induced by *E. coli* lipopolysaccharide and restored the epithelial barrier in a cell culture model, and alleviated the inflammatory response of colitis in mice (40, 56). *Alistipes* can also protect against certain diseases, including liver fibrosis, colitis and cardiovascular diseases (57). The abundance of *Alistipes*, particularly *Alistipes shahii*, was increased in the cohort of mice receiving the probiotic in the context of anti-inflammatory effects (58). In mice with colitis, the abundance of *Alistipes finegoldii* was significantly reduced, and colitis was significantly relieved by gavage with *A. finegoldii* (51). Several strains of *Bacteroides*, including *Bacteroides cellulosilyticus* DSM 14838, *B. fragilis* NCTC 9343, *B. ovatus* ATCC 8483, *B. thetaiotaomicron* DSM 2079 and ATCC29148, *B. vulgatus* ATCC 8482, mpk and 7K1 could inhibit inflammation in colitis by altering or blocking the production of cytokines (2, 9, 11, 12, 59, 60). Animal studies of colitis had demonstrated that *B. fragilis* NCTC 9343, *B. thetaiotaomicron* DSM 2079, and *B. cellulosilyticus* DSM 14838 improved intestinal histopathological injury and increased anti-inflammatory cytokine IL-10 and Treg cells levels by increasing the number of CD4^+^ CD45RB^low^ T cells (2, 59, 60). *B. vulgatus* 7K1 ameliorated DSS-induced colitis in mice by decreasing the concentrations of TNF-α and IL-6 and increasing the production of IL-10 in mouse colon tissues (12). Our data showed that *B. vulgatus* Bv46 treatment significantly increased the abundance of *B. vulgatus* in the feces of mice, and reduced the expression of colonic TNF-α, IL-6 and IL-1β.

Our data suggest that *B. vulgatus* Bv46 improved DSS-induced mouse colitis by regulating immune response, especially, B cell immune response. Studies have shown that hyperactive B cell immune response can aggravate the severity of ulcerative colitis (61, 62). In this study, *B. vulgatus* Bv46 significantly downregulated the expression of *Ccl19, Cd19, Cd22, Cd40* and *Cxcr5* genes, which mainly participate in the regulation of B-cell responses (63–65). CD40 activation is a key driver of B cell activation and CD40 is involved in the pathogenesis of IBD (65, 66). Immunohistochemical staining showed that CD40 staining in colon tissues of IBD patients was stronger than that of healthy individuals (67). CD40 overexpression was positively correlated with IBD (67). Microcystin-leucine arginine (MC-LR) exacerbated colitis through CD40 and CD40 knockout effectively attenuated the role of MC-LR in mice with pre-existing colitis (66). CD40 mediated CXCR5 expression in B cells, partially through activation of noncanonical nuclear transcription factor kappa B (NF-κB) pathway (68).

CD40 plays an important role in the activation of NF-κB which is up-regulated in IBD (69). NF-κB has been identified as one of the key regulators in activating proinflammatory cytokine production (70). NF-κB signaling occurs through both canonical and noncanonical pathways (71). Activation of the canonical NF-κB signaling cascade leads to transcription of a range of well-characterized inflammatory mediators, such as IL-1β, TNF-α, and IL-6 (72). In the noncanonical signaling cascade, NF-κB initiates the transcription of chemokines, including CXCL12, CXCL13, CCL19, and CCL21 (73). In IBD patients, NF-κB is activated and the increased expression of NF-κB in mucosal macrophages is accompanied by an increased ability of these cells to produce and secrete TNF-α, IL-1, and IL-6, thereby promoting the expression of various proinflammatory genes and influencing the process of mucosal inflammation (71). This study showed that compared with the control group, DSS treatment increased the expression of *Cd40* and *Ccl19* genes, while *B. vulgatus* Bv46 treatment reversed this effect of DSS. Moreover, *B. vulgatus* Bv46 treatment reduced the expression of colonic TNF-α, IL-6 and IL-1β in the DSS-induced mouse colitis *in vivo* and the TNF-α, IL-6 and IL-1β release in the supernatants of LPS-activated macrophage cells *in vitro*. These results imply that *B. vulgatus* Bv46 may alleviate DSS-induced mouse colitis by reducing the activity of NF-κB.

SCFAs, the main metabolites produced by bacterial fermentation of dietary fiber, play an important role in maintaining intestinal health (74) and are typically reduced in the mucosa and feces of IBD patients compared with healthy individuals (47, 75). SCFAs have been reported to inhibit the expression of LPS-induced cytokines such as IL-6 and TNF-α, and showed a strong anti-inflammatory effect (76–79). In the present study, *B. vulgatus* Bv46 produced SCFAs and significantly reduced the secretion of TNF-α, IL-1β and IL-6 in LPS-stimulated macrophages *in vitro*. This result suggests that SCFAs secreted by *B. vulgatus* Bv46 may inhibit the secretion of TNF-α, IL-1β and IL-6 by macrophages stimulated by LPS *in vitro.* Microbiota-derived SCFAs play an essential role in anti-inflammatory responses (80). In agreement with other studies (81–85), the abundance of SCFAs-producing bacteria, including *Parabacteroides*, *Bacteroides, Anaerotignum* and *Alistipes*, were increased in the feces of DSS-induced colitis mice treated with *B. vulgatus* Bv46. Oral administration of *B. vulgatus* Bv46 notably increased the contents of fecal SCFAs (including propionic acid, butyric acid, isobutyric acid, valeric acid and isovaleric acid) in DSS-induced colitis mice, which was in accordance with other studies (12). These findings suggest that *B. vulgatus* Bv46 treatment may relieve DSS-induced mouse colitis by increasing the abundance of gut microbiota producing SCFAs. It is well established that butyric acid plays a key role in protection against the development of IBD, and is mainly involved in the suppression of NF-κB in activated B cells and reinforcement of the colonic defense barrier (86, 87). Herein, the elevated level of butyric acid arising from *B. vulgatus* Bv46 gavage as detected in the feces may contribute to the alteration of the transcription of NF-κB. However, the specific mechanism needs to be further explored.

## Conclusion

This study showed that *B. vulgatus* Bv46 had a protective effect against DSS-induced mouse colitis by regulating gut microbiota and the contents of SCFAs, inhibiting the expression of colonic proinflammatory cytokines TNF-α, IL-6 and IL-1β and downregulating the expression of *Ccl19, Cd19, Cd22, Cd40* and *Cxcr5* genes. However, the specific mechanism by which *B. vulgatus* Bv46 interacts with the host to exert therapeutic effects requires further investigation.

## Data availability statement

The datasets presented in this study can be found in online repositories. The names of the repository/repositories and accession number(s) can be found below: https://www.ncbi.nlm.nih.gov/, PRJNA872285; https://www.ncbi.nlm.nih.gov/, PRJNA872866.

## Ethics statement

The animal study was reviewed and approved by The Ethics Review Committee of the National Institute for Communicable Disease Control and Prevention at the Chinese Center for Disease Control and Prevention (Beijing, China).

## Author contributions

LL, ZR and JX designed the project. JY performed the sampling. LQ, MX, XPL, SZ, XYL carried out the experiments. LL, MX and DH analyzed the data. LL, RL and JX drafted the manuscript. All authors contributed to the article and approved the submitted version.

## Funding

This work was supported by National Key R&D Program of China (2019YFC1200505 and 2019YFC1200500).

## Conflict of interest

The authors declare that the research was conducted in the absence of any commercial or financial relationships that could be construed as a potential conflict of interest.

## Publisher’s note

All claims expressed in this article are solely those of the authors and do not necessarily represent those of their affiliated organizations, or those of the publisher, the editors and the reviewers. Any product that may be evaluated in this article, or claim that may be made by its manufacturer, is not guaranteed or endorsed by the publisher.
