# Supplementary material for: Bacteroides vulgatus attenuates experimental mice colitis through modulating gut microbiota and immune responses
Source: Front Immunol. 2022 Nov 30;13:1036196. doi: 10.3389/fimmu.2022.1036196 (PMC9750758; doi:10.3389/fimmu.2022.1036196)
Supplement: Supplementary file 2 [file DataSheet_1.pdf]

***Bacteroides vulgatus* attenuates experimental mice colitis through modulating gut microbiota and immune responses**

Liyun Liu[1,3†], Mingchao Xu[1,2†], Ruiting Lan[4†], Dalong Hu[4], Xianping Li[1], Lei Qiao[1], Suping Zhang[1,2], Xiaoying Lin[1,2], Jing Yang[1,3], Zhihong Ren [1,3*] and Jianguo Xu[1,2,3,5*]

[1]State Key Laboratory of Infectious Disease Prevention and Control, National Institute for Communicable Disease Control and Prevention, Chinese Center for Disease Control and Prevention, Beijing 102206, China.

[2]Department of Epidemiology, Center for Global Health, School of Public Health, Nanjing Medical University, Nanjing 211166, Jiangsu Province, China.

[3]Research Units of Discovery of Unknown Bacteria and Function, Chinese Academy of Medical Sciences, Beijing 102206, PR China.

[4]School of Biotechnology and Biomolecular Sciences, University of New South Wales, Sydney, New South Wales, Australia.

[5]Institute of Public Health, Nankai University, Tianjin, PR China

* Corresponding author

E-mail addresses: xujianguo@icdc.cn (J. Xu), renzhihong@icdc.cn (Z. Ren).

†These authors contributed equally to this work

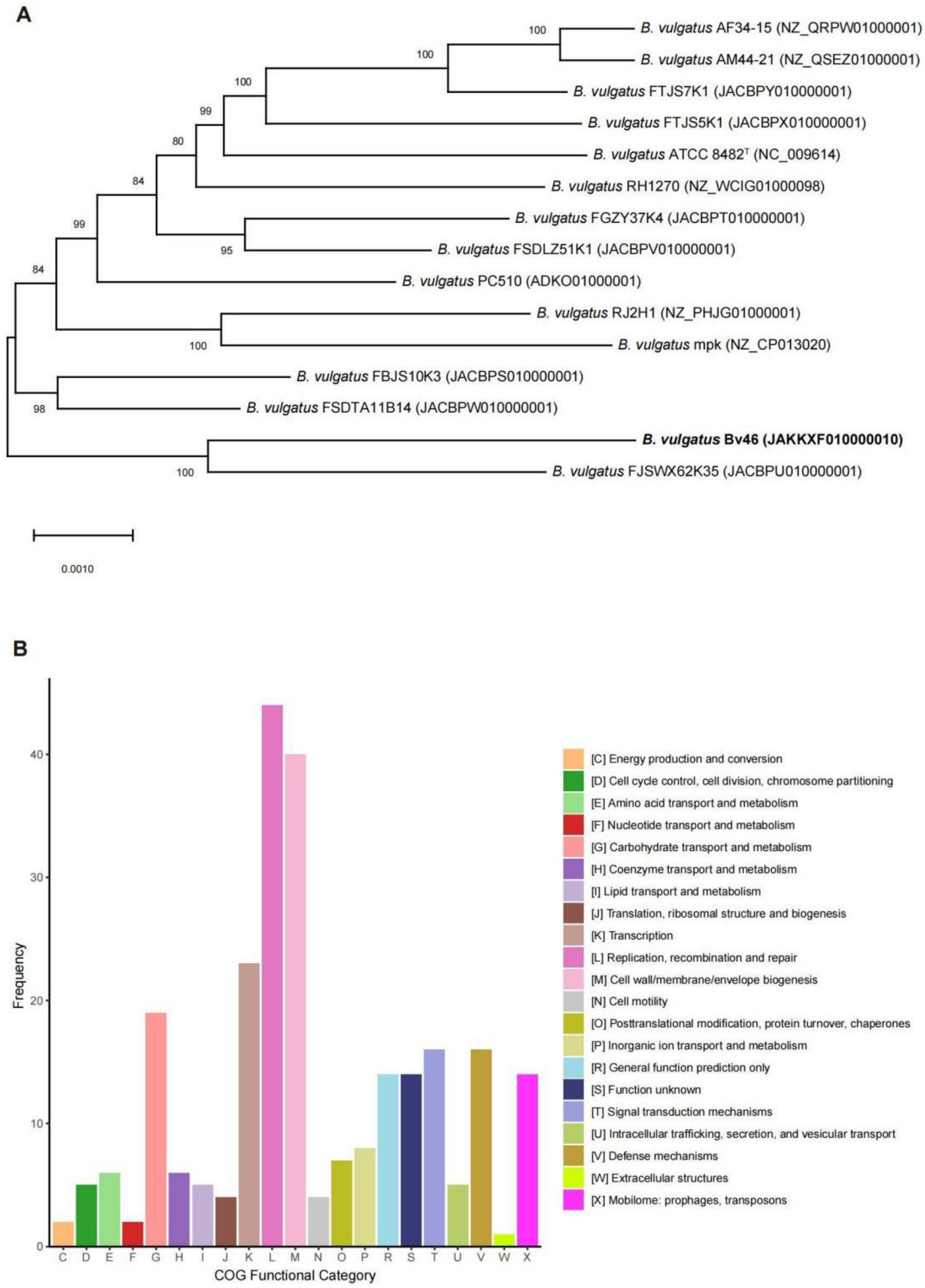

FIGURE S1 Genome analysis of *B. vulgatus* strains. (A) The phylogenetic relationship of 15 *B. vulgatus* strains based on core genes. (B) The number of *B. vulgatus* Bv46 strain-specific genes assigned in COGs (Clusters of Orthologous Groups) categories.

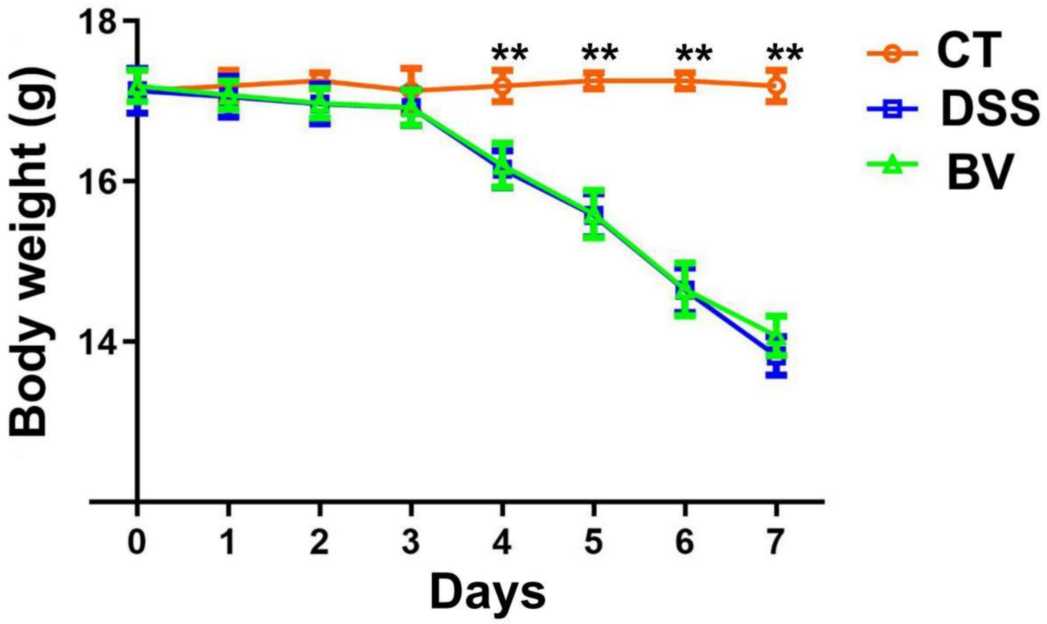

FIGURE S2 Changes in body weight of DSS-induced mice treated with *B. vulgatus* Bv46 (in BV group), or phosphate buffer saline (in DSS group), and control (CT, no DSS induction) for 7 days. Values represent the mean ± standard deviation. **, $P < 0.01$ indicated the significant differences of DSS group versus control group.

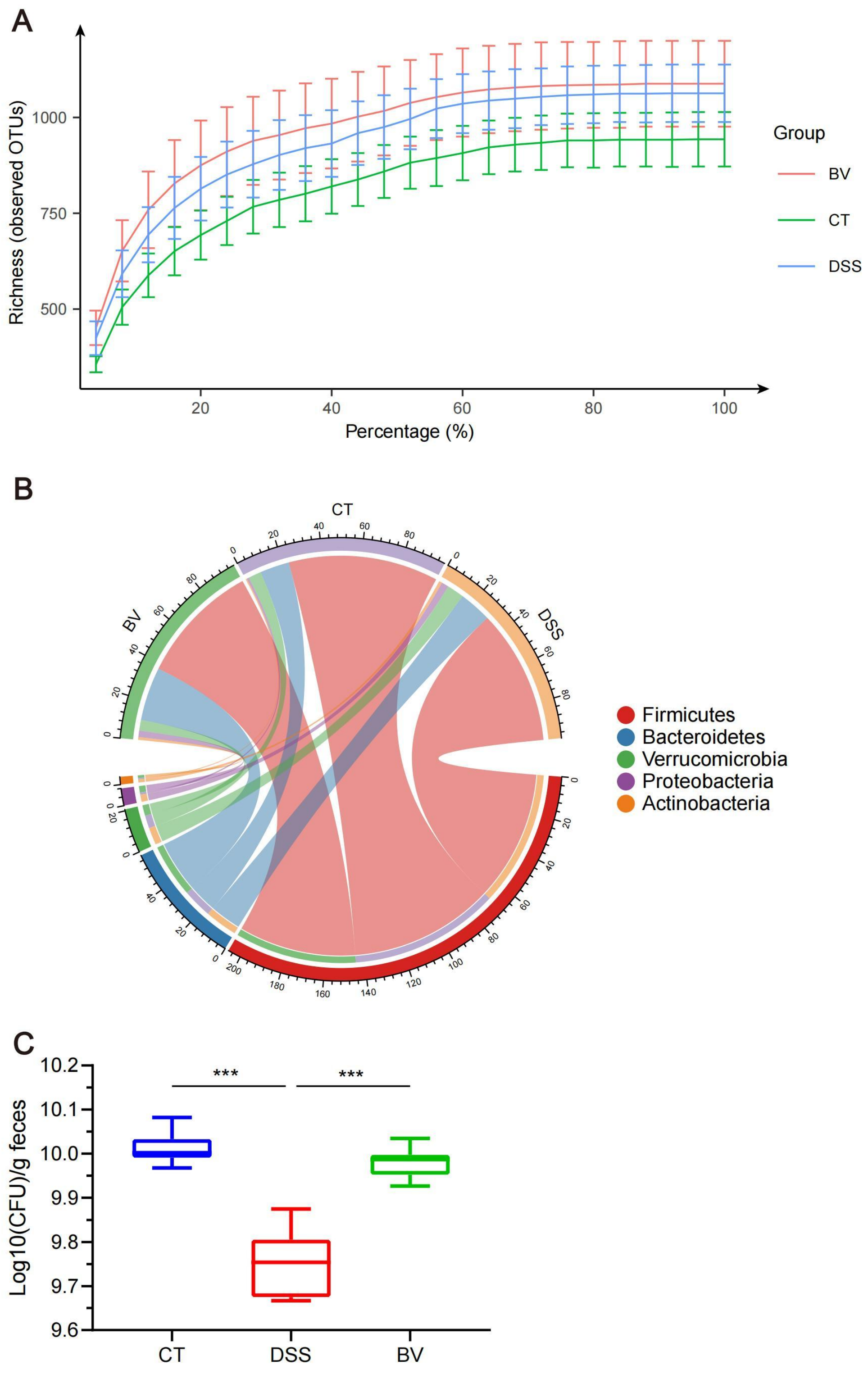

FIGURE S3 (A) Rarefaction curve analysis of observed OUTs from the three groups based on random sampling. (B) Overview of the dominant bacterial phyla in the three groups. (C) Species specific qPCR-tracked *B. vulgatus* in feces of mice. Statistical comparison was implemented by a one-way ANOVA followed by Tukey's multiple-comparison test. Values are expressed as quartiles in each group, ***, $P < 0.001$.

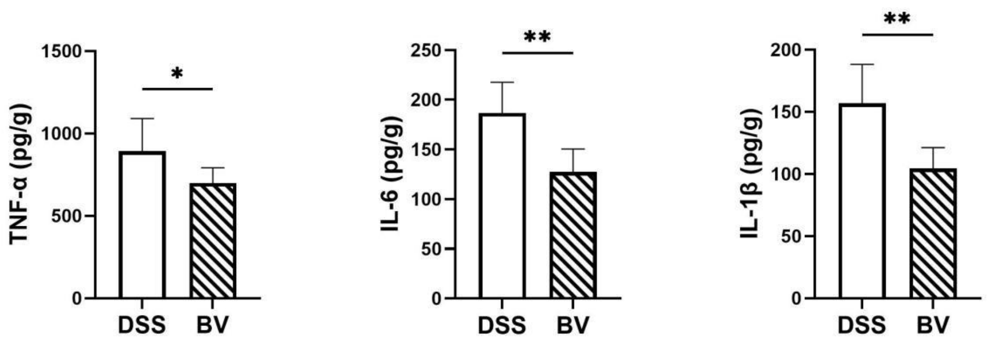

FIGURE S4 The expression of colonic inflammatory cytokines (TNF-α, IL-6 and IL-1β) in DSS and BV groups. Values represent the mean ± standard deviation. Significance levels are shown as *, $P < 0.05$; **, $P < 0.01$.
